# Development and Analysis of the Novel Hybridization of a Single-Flash Geothermal Power Plant with Biomass Driven sCO_2_-Steam Rankine Combined Cycle

**DOI:** 10.3390/e23060766

**Published:** 2021-06-18

**Authors:** Balkan Mutlu, Derek Baker, Feyza Kazanç

**Affiliations:** 1 Department of Mechanical Engineering, Middle East Technical University, Ankara 06800, Turkey; dbaker@metu.edu.tr (D.B.); fkazanc@metu.edu.tr (F.K.); 2 Center for Solar Energy Research and Applications (GÜNAM), Ankara 06800, Turkey

**Keywords:** hybridization, single-flash, geothermal, biomass, sCO_2_ cycle, olive residue, flexibility

## Abstract

This study investigates the hybridization scenario of a single-flash geothermal power plant with a biomass-driven sCO_2_-steam Rankine combined cycle, where a solid local biomass source, olive residue, is used as a fuel. The hybrid power plant is modeled using the simulation software EBSILON^®^Professional. A topping sCO_2_ cycle is chosen due to its potential for flexible electricity generation. A synergy between the topping sCO_2_ and bottoming steam Rankine cycles is achieved by a good temperature match between the coupling heat exchanger, where the waste heat from the topping cycle is utilized in the bottoming cycle. The high-temperature heat addition problem, common in sCO_2_ cycles, is also eliminated by utilizing the heat in the flue gas in the bottoming cycle. Combined cycle thermal efficiency and a biomass-to-electricity conversion efficiency of 24.9% and 22.4% are achieved, respectively. The corresponding fuel consumption of the hybridized plant is found to be 2.2 kg/s.

## 1. Introduction

The performance of geothermal power plants can degrade over the years, as the geothermal resource is exploited. This degradation can be due to decreases in fluid temperature, flow rate, and pressure at the production wellhead over the lifetime of the geothermal resource. These decreases can reduce both the quantity (thermal energy) and quality (temperature) of the heat input, and lead to reductions in both the output and the thermal efficiency of the plant [1]. The performance of geothermal power plants is also typically negatively affected by increases in ambient temperatures, especially during the hot summer months. The power fluctuations due to the changes in ambient temperatures can exceed 20% in some scenarios [2]. The hybridization of geothermal resources with other renewable thermal energy resources offers the potential to increase the performance of geothermal power plants while being economically feasible, since the need for land and grid infrastructure can be reduced or eliminated. However, the hybridization of geothermal energy with other renewable thermal energy resources is a site-specific matter, due to the site-specific nature of the geothermal energy itself [3]. In the context of this article, Kızıldere-1 (KZD-1), an existing single-flash Geothermal Electric Power Plant (GEPP), operating significantly below design capacity, is considered as a case study for hybridization with biomass energy. KZD-1 is located on the Western Anatolia region of Turkey, where Olive Residue (OR) exists as an abundant solid biomass resource. The hybridization scenario in this article concentrates on bringing the underutilized KZD-1 GEPP to its full capacity through the use of thermal heat derived from this local biomass source. The colocation of geothermal and biomass as a secondary renewable thermal energy source is exploited through utilizing the unused capacity of KZD-1 turbine and introducing a next-generation power cycle, i.e., supercritical CO_2_ (sCO_2_) cycle, as a topping cycle. A sCO_2_ cycle is chosen for its potential to support flexible electricity generation. The article is structured to allow the method and outcomes to be adapted to other GEPP.

The interest in the hybridization of geothermal with biomass is limited in the literature. The state-of-the-art studies are limited to those using biomass-sourced heat to supplement the operating enthalpy of low-temperature geothermal [4], using biomass to compensate for the off-design conditions of a geothermal power plant [5], and multigeneration biogas–geothermal systems [6]. When the colocation of geothermal and agricultural lands or forestry are present, either biomass or geothermal is used to boost the performance of the other for actual industrial applications. For instance, a dry steam geothermal power plant in Cordia, Italy, operating below its rated output of 20 MWe, is redesigned to accommodate a biomass furnace in order to superheat the geothermal steam. An additional 5.4 MWe gain is obtained from the biomass combustion. In another example, a wood waste biomass power plant operating in close proximity to forest plantations in New Zealand is supplemented by a geothermal preheat [7].

Despite the limited studies on the hybridization of geothermal with biomass, the literature on hybridization of flash-type geothermal power plants with solar thermal is more developed [8] and can provide insight into the design of hybrid geothermal–biomass power plants. Solar thermal heat is used in most of these studies, either to preheat or superheat the geothermal working fluid. Initial efforts on hybrid solar–geothermal power plant models are by Lentz and Almanza [9,10], where a Direct Steam Generation (DSG) solar field is theoretically coupled to two different locations of the Cerro Prieto geothermal single flash plant in Mexico. In both of their proposed models, geothermal brine is allowed to pass through the tubes of a Parabolic Trough Collector (PTC) field with the purpose of increasing the flow rate of geothermal steam by 10%. Mir et al. [11] developed a thermodynamic model to estimate the production of a hypothetical solar–geothermal power plant in Northern Chile for two different operational modes: (1) Peak Power mode with constant geothermal output where the solar input increases the power output; (2) Save Geothermal Resource mode with constant power output, and the use of geothermal resources decreases with increases in solar input. Note that, although individual authors often used different names for these modes, this naming is used for clarity and consistency within this section. Mir et al. [11] added solar heat to a single-flash geothermal power plant from a PTC field to produce superheated steam and additional saturated steam from the separator whenever possible. With the assistance of solar heat, up to an 11.6% increase in energy production from the geothermal brine is obtained for the Peak Power mode, whereas savings of up to 10.3% in the use of geothermal resources is obtained for the Save Geothermal Resource mode. Cardemil et al. [12] conducted energetic and exergetic analyses for hypothetical single- and double-flash geothermal power plants, with each having four different brine characteristics, taking Save Geothermal Resource and Peak Power modes into account. A PTC field is used to supply solar heat to the system after the separator, both to the geothermal brine and as additional steam and superheat, respectively. Their results show that a hybrid single-flash power plant can produce at least 20% additional power output, depending on the brine characteristics. For all the analyzed cases, at least a 3% increase is observed for first law efficiencies. For the Save Geothermal Resource mode, where the plant power output stays constant when solar resources are available, 16% and 19% reductions in geothermal fluid consumption are observed for flash and double-flash configurations, respectively. A more recent and comprehensive study on the hybridization of flash-type geothermal power plants is conducted by McTigue et al. [13]. In their study, an existing double-flash power plant operating in China Lake, California, is hybridized with solar thermal to increase the power generation. It aimed to increase the power output of the geothermal turbine operating at 75% of its rated output (22.5 MW) to 100% of its rated output (30 MW) by integrating an array of PTCs. In order to decide the optimum point for solar thermal heat addition from the PTC heat transfer fluid to the double-flash geothermal power plant, parametric analyses of thermal efficiency are compared for four different points within the double-flash GEPP. The optimum point for heat addition is found to be the brine after the first flash tank separator. The solar heat added to this brine is converted to electricity with an efficiency of 24.3%. Alternate thermal storage scenarios are also investigated for dispatchable electricity generation. Overall, the Levelized Cost of Electricity (LCOE) with 3 h of thermal energy storage is calculated as 0.08 $/kWhe, which is lower than the equivalent conventional standalone CSP and battery integrated PV systems. It is concluded that hybridization can be cost-effective, since the existing power block, pipework, and condenser are shared.

A novel hybrid solar integration for a binary-type geothermal plant has been proposed by Bonyadi et al. [14]. Although binary plants have different characteristics to flash type plants, their introduction of a topping cycle for hybridization formed the inspiration for the efforts in this article. Bonyadi et al. [14] added a solar-powered steam Rankine topping cycle to a hypothetical binary geothermal power plant, without requiring any physical modification or deviation from design conditions of the bottoming Organic Rankine Cycle (ORC). The solar topping cycle is coupled to the ORC so that the waste heat from the topping cycle is utilized in the ORC. Their representative design has an incremental solar efficiency of 12.2% for Peak Power mode and consumes up to 17% less brine than a similar stand-alone geothermal plant for the Save Geothermal Resource mode.

For the completely different technology of Enhanced Geothermal System (EGS), Jiang et al. [15] integrated solar thermal heat into a hypothetical EGS where CO_2_ is used as both heat transmission fluid for geothermal and working fluid for the sCO_2_ power cycle. Their hybrid system uses geothermal energy as the primary energy source to provide the base-load electricity, and the solar energy is used as a supplement to meet the peak demand whenever possible. Their hybrid plant reaches the maximum thermal efficiency of 22.44% for a CO_2_ turbine inlet temperature of 600 °C.

The sCO_2_ cycle studied in this article is a part-flow type sCO_2_ cycle. Despite the equivalent or higher thermodynamical efficiencies of sCO_2_ cycles compared to their steam Rankine counterparts, there has not been a full-scale commercial demonstration of the sCO_2_ cycles as the studies are limited to laboratory-scale test setups under 1 MW [16,17,18,19]. The underlying reason for sCO_2_ cycles offering good thermal efficiency is that the compression work of CO_2_ as a working fluid close to its critical point of 31.1 °C and 7.39 MPa is minimal [20]. However, thermophysical properties of CO_2_, such as the isobaric heat capacity in the vicinity of its critical point, exhibit non-linear behavior and result in a pinch-point problem. Utamura [21] demonstrated that a first law efficiency of 45% under maximum operating conditions of 20 MPa and 526.9 °C is achievable for part-flow sCO_2_ cycles where the pinch-point problem can be avoided. The part-flow configuration helps to confine the likelihood of pinch-point problems to the low-temperature recuperator (LTR) by splitting the rest of the recuperation process to a high-temperature recuperator (HTR). Overall, part-flow sCO_2_ cycles can offer a more than 5% increase in the thermal efficiency compared to simple recuperated sCO_2_ cycles, and are the most extensively researched sCO_2_ cycle in the literature, as they are relatively simple and retain good efficiency [22]. In addition to the pinch-point problem, the sCO_2_ cycles have another intrinsic problem regarding the heat addition to the cycle. Due to their highly recuperative characteristics, the external heat addition to sCO_2_ cycles is carried out over a high-temperature interval [23,24,25]. To overcome this limitation, the sCO_2_ cycles are often combined with bottoming ORC cycles operating at low temperatures [26,27,28,29], or utilized in cascading manner as sCO_2_–sCO_2_ and sCO_2_–transcritical carbon dioxide (tCO_2_) cycles [23,24,25]. Alternatively, for coal-powered sCO_2_ cycle designs, advanced boiler and heater designs are introduced to fully exploit the available heat in the flue gas within the cycle, at the cost of obtaining more complex layouts [30,31]. Motivated by the problem of a biomass-powered sCO_2_ cycle design, Manente and Lazzaretto [32] introduced a novel cascaded sCO_2_ cycle configuration using woody biomass as a fuel. In their study, two different cascaded sCO_2_ cycles, namely part-flow–simple-recuperated and simple-recuperated–simple-recuperated, are investigated, along with four different biomass boiler arrangements. Their results showed that part-flow–simple-recuperated cascaded sCO_2_ cycle design with a counter-current radiative–convective boiler demonstrated the best performance in terms of biomass-to-electricity conversion efficiency, i.e., either 34% or 36%, depending on the presence of an air-preheating unit, for the topping cycle turbine inlet temperature (TIT) of 550 °C.

The work in this article aims to build on this existing literature by presenting a novel scheme to hybridize an existing single-flash GEPP with biomass-derived heat that can be adapted to any site where colocation of these two renewable resource types is present. KZD-1 is used as a case study to apply this novel method and analyze its performance.

## 2. Novel Hybrid Geothermal–Biomass Power Plant Scheme

Based on the reviewed literature, for an existing flash type GEPP suffering from a degradation in its geothermal resource in terms of a decreased flow rate or enthalpy, which ultimately causes a reduced steam flow to its turbine, the following novel hybrid geothermal-biomass hybridization scheme can be adapted if colocated with a solid biomass resource. A part-flow sCO_2_ cycle, to be operated at a higher temperature than the existing geothermal cycle, can be used as a topping cycle. The part-flow sCO_2_ cycle can reject its waste heat through its cooler to preheat the condensate of the existing geothermal cycle. A novel biomass heater-boiler designed for this unique application can supply high-temperature heat from biomass combustion through radiative heat transfer to drive the part-flow sCO_2_ topping cycle, and bring the preheated geothermal condensate to a certain steam quality. The medium-temperature heat of the flue gas is transferred to this steam-water mixture by means of convective heat transfer to create dry steam. This 100% biomass-energy-derived dry steam can be fed to the steam turbine of an existing GEPP operating under capacity due to a reduction in its mass flow. Moreover, when appropriate, the existing unused cooling component capacity of GEPP can be used to condensate the biomass derived steam exhaust for better utilization. Such a novel hybridization scheme offers several advantages. First, the rejected heat of the topping sCO_2_ cycle is not lost, but instead used to supply heat for an additional dry steam. Second, sCO_2_ cycles are either utilized in a cascaded manner [23,24,25,32] or combined with ORC bottoming cycles operating at low temperatures [26,27,28,29] due to their high-temperature heat requirement. Since the medium temperature heat of the flue gas is used for the creation of additional dry steam, the need to use an additional bottoming ORC or sCO_2_ cycle is eliminated. As a result, the existing GEPP can be brought to full capacity to allow for better Capex utilization. Note that only the unused flow capacity of steam turbine and, if possible, an existing cooling system is used for such a hybridization scenario, while operational steam turbine inlet conditions, i.e., pressure and temperature, remain unchanged. In this sense, hybridization is possible without modifying the components of the existing GEPP.

### 2.1. Application of Proposed Novel Hybridization Scheme to KZD-1 GEPP

#### 2.1.1. Existing Conditions of KZD-1 GEPP

KZD-1 GEPP is the first geothermal power plant in Turkey, commissioned in 1984 and currently operated by Zorlu Energy [33]. The steam mass flow rate feeding the steam turbine of KZD-1 decreased significantly over the years, considering the differences between the current mass flow rate of 19.45 kg s^−1^ and the average steam mass flow rate of 33.34 kg s^−1^ reported by Gökçen et al. in 2004 [34]. Moreover, the steam turbine of KZD-1 seems to have been worn out over its active years, considering its current calculated isentropic efficiency of 30% and reported isentropic efficiencies of 71.2% and 71.5% in the literature [33,34]. In order to better represent single-flash GEPPs and make the hybridization efforts meaningful, within the context of this article, the isentropic efficiency of the steam turbine is assumed to be 80%, in line with the typical isentropic efficiencies of geothermal steam turbines suggested by DiPippo [33].

**Figure 1:**
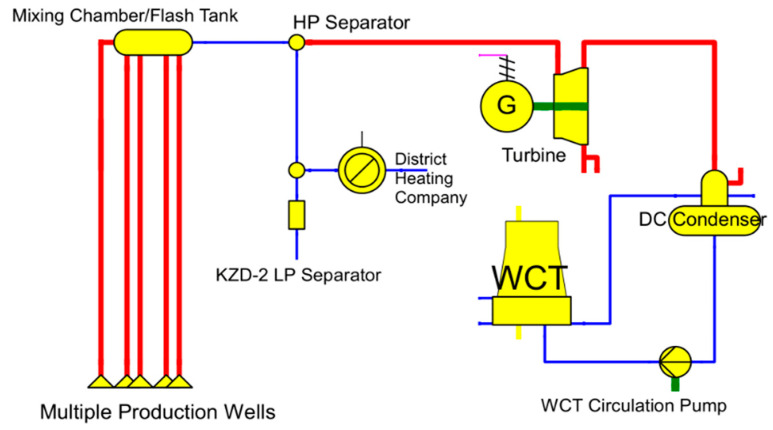
Existing schematic of KZD-1 GEPP, as drawn in EBSILON^®^Professional.

KZD-1 is a typical single-flash GEPP with multiple production wells; its schematic is supplied in Figure 1. Although a non-condensable gas (NCG) extraction system exists in reality, it is excluded within the context of this article in order to focus on thermodynamic modeling. After the two-phase geothermal brines were collected and flashed to 0.438 MPa and 146.9 °C, the geothermal brine and steam were separated through the high-pressure (HP) separator. The geothermal brine was used for district heating before being directed to the neighboring Kızıldere-2 (KZD-2) GEPP’s low-pressure (LP) separator, where it was ultimately re-injected. The geothermal steam after the HP separator reached 146.91 °C, with a flow rate of 19.45 kg s^−1^; it then passed through the steam turbine and the exhaust steam was condensed through the direct contact (DC) steam condenser. The condensed steam was then pumped to the wet cooling tower (WCT), where it was used as a cooling water and ultimately evaporated. The WCT used no make-up water, since the geothermal condensate was used as cooling water.

#### 2.1.2. Biomass Fuel Source

KZD-1 GEPP is located on the border of two western Anatolian cities of Turkey, Aydın and Denizli. In this region, the olive oil sector is well-developed [35]. Olive residue (OR) is a by-product of olive oil production and found abundantly in the region, where it is mostly used for domestic heating [36]. To exploit the synergistic colocation of the two renewable energy resources, OR samples from a nearby olive oil factory, KZD-1 GEPP, were collected. The results of the conducted analyses of the OR are presented in Table 1.

The higher heating value (HHV) of the biomass fuel was calculated based on its ultimate analysis following the procedure supplied by Sheng and Azevedo [37]. A lower heating value (LHV) was calculated from the HHV, in parallel with the work of Manente and Lazzaretto [32]. As a cross-check for HHV, Magãlhaes et al. [38] reported the HHV of their OR sample collected from the Balıkesir region of Turkey as 20.11 MJ kg^−1^, which is in good agreement with the calculated HHV for the OR samples used in this article. The ultimate analysis and heating values of the biomass fuel were used as inputs to simulation software EBSILON^®^Professional where the hybrid power plant is modeled.

#### 2.1.3. Model Development

**Figure 2:**
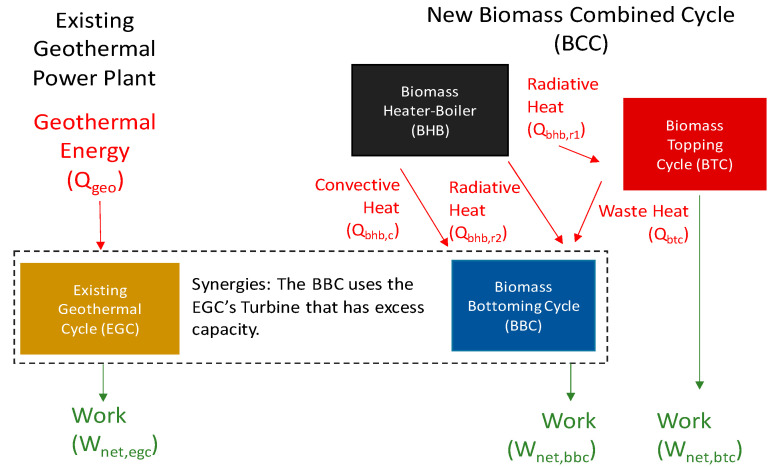
Thermodynamic conceptualization of proposed hybrid power plant.

A thermodynamic model was developed using EBSILON^®^ Professional software for the application of the proposed novel hybridization scheme to KZD-1 GEPP. The thermodynamic concepts of the associated model are highlighted in Figure 2. The proposed hybrid scheme consists of the following three cycles:

1. BTC: Biomass combustion driven sCO_2_ Topping Cycle;
2. BBC: Bottoming Biomass combustion and topping cycle waste, heat-driven steam Rankine Cycle;
3. EGC: Existing open-loop steam Rankine cycle, driven by geothermal energy (EGC).

Here, a combination of BTC and BBC forms the new biomass combined cycle (BCC). Note that the conceptualization in Figure 2 is strictly for thermodynamic modeling and enables the thermal efficiencies for the BCC, BTC, and BBC to be calculated.

**Figure 3:**
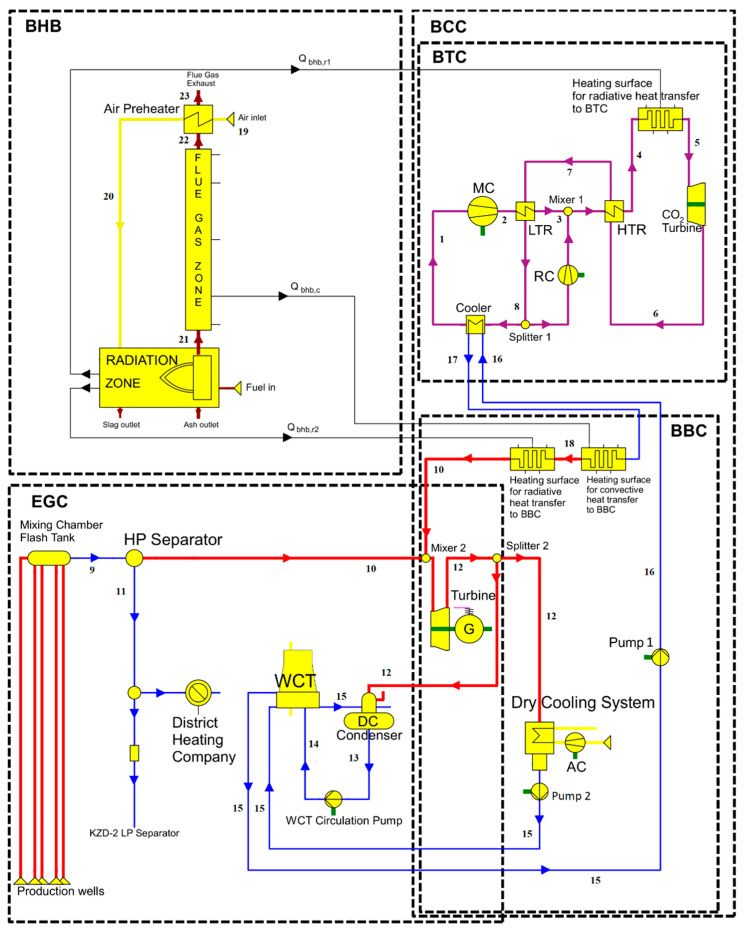
Schematic of the developed hybrid plant in EBSILON^®^Professional.

In terms of thermodynamic modeling, the most prominent feature of the existing KZD-1 GEPP is that it utilizes an open-loop (not cyclic) steam Rankine cycle, since the condensate outlet of the DC condenser is used as cooling water in WCT and eventually lost due to evaporation. The flow rate of this lost cooling water is equal to the original geothermal steam, separated by the HP separator, which then passes through the steam turbine of KZD-1, as shown in Figure 1. Due to this condition of the KZD-1 GEPP, sharing of the unused capacity of the existing cooling system is specifically avoided. In other words, the existing cooling system of KZD-1 is not used, to avoid the eventual loss of biomass-derived dry steam through the WCT. For this purpose, a hypothetical steam Rankine bottoming cycle (BBC) was introduced to form a closed-loop biomass combined cycle (BCC) along with the biomass topping cycle (BTC). Although the hypothetical BBC and EGC are shown separately in Figure 2, they are not physically separate, as both cycles share the existing steam turbine of KZD-1. Specifically, BBC is a theoretical construct to model a closed-loop BCC. While BTC rejects its waste heat to BBC, as shown in Figure 2, heat rejection from BBC is to the surroundings and achieved by means of a hypothetical dry cooling system, shown in the overall hybrid power plant layout in Figure 3.

If EGC were to be a closed-loop cycle, there would be no need to construct the hypothetical BBC, since EGC could be used as the bottoming cycle. Overall, application of the proposed novel hybridization scheme to KZD-1 GEPP resulted in two hypothetical cycles, i.e., BTC and BBC, whose combination constitutes a fully biomass-driven, closed-loop combined cycle, BCC. The configuration of the hybrid plant is presented in Figure 3.

The topping part-flow sCO_2_ cycle used for hybridization (BTC) was adapted from the work of Utamura [21]. The characteristics of the optimized BTC are presented in Table 2. The turbine inlet condition is fixed at 550 °C and 20 MPa to be parallel with the part-flow sCO_2_ cycles designs in the literature [32,39,40,41,42,43]. The heat rejection from BTC was transferred the BBC through the cooler. Heat input to BTC was achieved by means of radiative heat transfer from the biomass heater-boiler (BHB).

Since the existing steam Rankine cycle, driven by geothermal energy (EGC), is open-loop and has the operating conditions mentioned in Section 2.1.1, a new, closed-loop, hypothetical steam Rankine cycle (BBC), driven by biomass heat and waste heat from BTC, was developed as follows. The working fluid, water at 0.1 MPa and 29 °C (State 15), was taken from the basin of the KZD-1 WCT and pumped to a slightly higher pressure than the steam turbine inlet pressure (State 16) to account for the subsequent pressure losses. Using the rejected heat from BTC via its cooler, water was sensibly preheated to a temperature of 127.7 °C (State 17), close to its saturation temperature of 146.9 °C. The preheated water at State 17 was heated with the radiation from the biomass combustion until it reached 40% steam quality (State 18) and was brought to dry steam phase (State 10) by the convective heat transfer from the flue gas. Then, it was mixed with the geothermal steam at Mixer 2 and allowed to pass through the steam turbine. After passing through the turbine, the biomass-sourced fraction of the exhaust steam was extracted through the Splitter 2 (State 12), condensed, and sensibly cooled via the hypothetical dry cooling system, before being pumped into the basin of the WCT at the same thermodynamic state it was originally taken from (State 15). The partial extraction of steam exhaust at Splitter 2 and its dry cooling were carried out to avoid water consumption and create a closed-loop steam Rankine cycle, BBC. EGC and BBC are not physically separate, as both cycles use the same steam turbine. The co-occurrence of these two cycles starts at Mixer 2 and lasts until the biomass-derived portion of the steam exhaust is extracted through Splitter 2 at State 12. BBC is used to provide additional dry steam to the steam turbine which, before hybridization, is operating under capacity. Since the heat supplied to BBC is purely derived from biomass, the additional power production of the steam turbine is due to the addition of dry steam and is attributed to the BBC.

**Figure 4:**
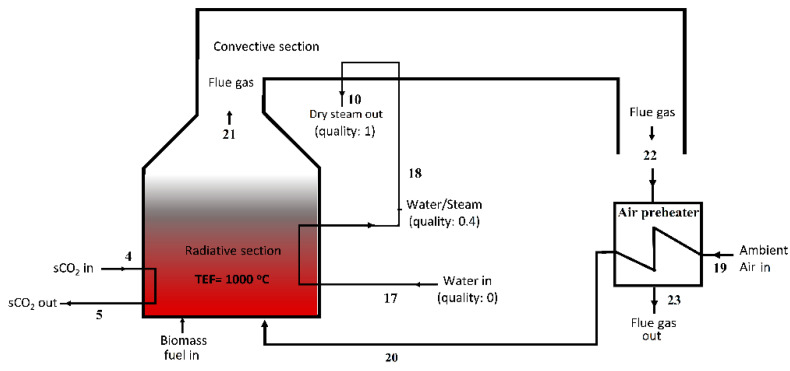
Radiative-convective counter-current heater-boiler (BHB) layout.

The radiative-convective, counter-current heater configuration from the work of Manente and Lazzaretto is modified and adapted for use in this article to drive both BTC and BBC [32]. Since the biomass combustion heat is used to add heat to the supercritical working fluid of BTC, and to create dry steam for BBC, this heating element is named the Biomass Heater and Boiler (BHB). An alternative schematic of the BHB is shown in Figure 4 with state numbers compatible with Figure 3. BHB can be considered as a discrete element from the cycles whose role is to supply the heat required by BTC and BBC. The radiation from the biomass combustion is used to add heat to BTC inside the radiative section of BHB. As described above, the working fluid of BBC, water, which was previously preheated through the BTC cooler, is brought to 40% steam quality using the radiation from the combustion in the radiative section of BHB. The water–steam mixture at 40% steam quality was then brought to the dry steam phase by means of convective heat transfer from the flue gas, before being mixed with the geothermal steam in Mixer 2. The remaining low-temperature useful heat in the flue gas was recovered using a counter-current air preheater. Finally, the flue gas was sent to the exhaust at 110 °C, which is greater than the dew point, to prevent condensation.

#### 2.1.4. Energy Analysis

The thermodynamic modeling of the proposed hybrid configuration in Figure 3 was performed using EBSILON^®^Professional software and its EbsBoiler module. The design conditions and characteristics of the components are presented in Table 2. Due to the novelty of the proposed hybrid configuration, the well-known energetic performance parameters are slightly different from their standalone definitions and are defined as follows.

The heat inputs to cycles shown in Figure 2 are defined sequentially as

(1) $${\;\dot{Q}}_{\mathrm{bhb},r1}={\;\dot{m}}_{4}\;\left(h_{5}-h_{4}\right)$$

(2) $${\;\dot{Q}}_{\mathrm{bhb},r2}={\;\dot{m}}_{17}\;\left(h_{18}-h_{1}\right)$$

(3) $${\;\dot{Q}}_{\mathrm{bhb},c}={\;\dot{m}}_{17}\;\left(h_{18}-h_{17}\right)$$

(4) $${\;\dot{Q}}_{\mathrm{btc}}={\;\dot{m}}_{17}\;\left(h_{17}-h_{16}\right)$$

where${\dot{\;Q}}_{\mathrm{bhb},r1}$ and ${\;\dot{Q}}_{\mathrm{bhb},r2}$ are the radiative heat transfer from BHB to BTC and BBC, respectively. While ${\;\dot{Q}}_{\mathrm{bhb},c}$ represents the convective heat transfer from BHB to BBC, ${\;\dot{Q}}_{\mathrm{btc}}$ represents the waste heat rejection from BTC cooler to BBC.

The net power outputs of the cycles are defined as

(5) $${\;\dot{W}}_{\mathrm{net},\mathrm{btc}}={\;\dot{W}}_{\mathrm{turb},\mathrm{btc}}-{\;\dot{W}}_{\mathrm{MC},\mathrm{btc}}-{\;\dot{W}}_{\mathrm{RC},\mathrm{btc}}$$

(6) $${\;\dot{W}}_{\mathrm{net},\mathrm{bbc}}={\;\dot{W}}_{\mathrm{turb},\mathrm{bbc}}-{\;\dot{W}}_{\mathrm{dry}\mathrm{cooling}\mathrm{sys},\mathrm{bbc}}$$

(7) $${\;\dot{W}}_{\mathrm{net},\mathrm{bcc}}={\;\dot{W}}_{\mathrm{net},\mathrm{bbc}}+{\;\dot{W}}_{\mathrm{net},\mathrm{btc}}$$

Consequently, the thermal efficiencies are calculated as

(8) $$\eta_{\mathrm{btc}}=\frac{{\;\dot{W}}_{\mathrm{net},\mathrm{btc}}}{{\;\dot{Q}}_{\mathrm{bhb},r1}}$$

(9) $$\eta_{\mathrm{bbc}}=\frac{{\;\dot{W}}_{\mathrm{net},\mathrm{bbc}}}{{\;\dot{Q}}_{\mathrm{bhb},r2}+{\;\dot{Q}}_{\mathrm{bhb},c}+{\;\dot{Q}}_{\mathrm{btc}}}$$

(10) $$\eta_{\mathrm{bcc}}=\frac{{\;\dot{W}}_{\mathrm{net},\mathrm{bcc}}}{{\;\dot{Q}}_{\mathrm{bhb},r1}+{\;\dot{Q}}_{\mathrm{bhb},r2\;}+{\;\dot{Q}}_{\mathrm{bhb},c}}$$

Note that ${\;\dot{Q}}_{\mathrm{btc}}$ is not present in the denominator of Equation (10) due to its representing an internal heat transfer within the combined cycle, BCC.

The biomass heater-boiler (BHB) efficiency, representing the efficiency of the heat transfer from chemical biomass energy to BCC, is defined in terms of a direct method as

(11) $$\eta_{\mathrm{bhb}}=\frac{{\;\dot{Q}}_{\mathrm{bhb},r1}+{\;\dot{Q}}_{\mathrm{bhb},r2}+{\dot{\;Q}}_{\mathrm{bhb},c}}{{\;\dot{m}}_{\mathrm{fuel}}{\;\mathrm{LHV}}_{\mathrm{fuel}}}$$

where ${\;\dot{m}}_{\mathrm{fuel}}$ and ${\mathrm{LHV}}_{\mathrm{fuel}}$ are the biomass fuel consumption and LHV of the biomass fuel, respectively.

Finally, the biomass-to-electricity conversion efficiency,${\;\eta }_{b2e}$, can either be found with the multiplication of $\eta_{\mathrm{bcc}}$ and $\eta_{\mathrm{bhb}}$, or explicitly from

(12) $$\eta_{b2e}=\frac{{\;\dot{W}}_{\mathrm{net},\mathrm{bcc}}}{{\;\dot{m}}_{\mathrm{fuel}}{\;\mathrm{LHV}}_{\mathrm{fuel}}}$$

#### 2.1.5. Model Verification

**Figure 5:**
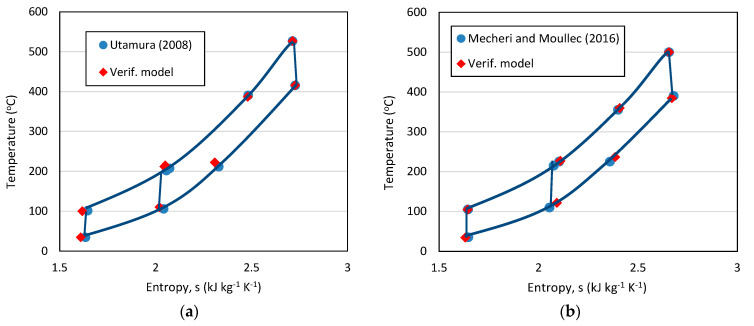
T-s diagrams of the standalone part-flow sCO_2_ cycle verification models against the models in the literature: (**a**) Verification against Utamura [21]; (**b**) Verification against Mecheri and Moullec [31].

Although it is not possible to verify the proposed hybrid power model due to its novelty, its constituting elements can be verified separately. The EGC model is verified using the actual operating power plant data supplied by Zorlu Energy. Two standalone part-flow sCO_2_ cycles are modeled in EBSILON^®^Professional using the design parameters supplied by Utamura [21] and Mecheri and Moullec [31]. T-s diagrams of the verification models are verified against the T-s diagrams supplied by Utamura [21] and Mecheri and Moullec [31] in Figure 5. The calculated first law efficiencies of 44.6% and 39% for the verification models of these two respective studies are in line with their reported values of 45% and 39%.

The verification of the radiative-convective BHB model was carried out by creating a verification combustion model in an EbsBoiler module of EBSILON^®^ Professional for the different cases presented in the study of Manente and Lazzaretto [32]. The verification results are presented in Table 3 for 1 kg s^−1^ woody biomass fuel used in their work. Note that the effective temperature of radiation (TEF) and the heat loss in the radiative section is assumed to be 1000 °C and 5%, respectively, throughout this article, to be consistent with Manente and Lazzaretto [32].

Considering the match in the T-s diagrams, consistency in thermal efficiencies, and the maximum absolute error of 2.3% in the BHB verification results, the proposed hybrid configuration in this article is assumed to be modeled accurately.

#### 2.1.6. Optimization of Input Parameters

For their sCO_2_ cycle design for waste heat recovery systems, Manente and Fortuna [47] state that one of the main novelties in the recent literature on hybrid plant layouts is the sharing of some equipment to reduce the number of components. The efforts in this article aim to use the existing infrastructure of the KZD-1 GEPP to the fullest extent through sharing the existing steam turbine of KZD-1 with EGC and BBC. Concurrently, hybridization scenarios where the design operating conditions of the existing KZD-1 GEPP are changed by means of an increased steam turbine inlet temperature or the generation of superheat steam through biomass combustion are avoided, as power plant operators are generally not willing to make changes to their design conditions. In this sense, the hybridization exploits the excess steam turbine capacity resulting from the degradation in mass flow of the geothermal steam over the years by using the dry steam derived from biomass combustion in BBC to partially return the steam turbine to its design operating conditions. The mass flow rate of this additional biomass-derived dry steam is equal to the mass flow rate of the BBC working fluid, water.

**Figure 6:**
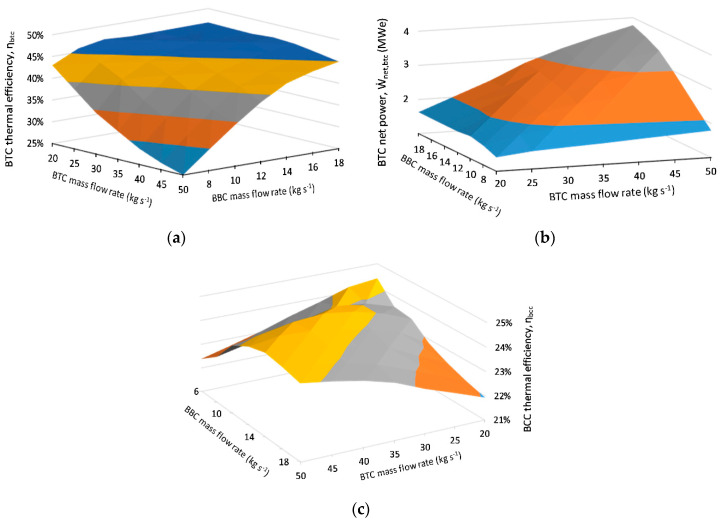
Results of parametric analysis conducted on the flow rates of BTC and BBC. (**a**) BTC thermal efficiency,${\;\eta }_{\mathrm{btc}}\;$; (**b**) BTC net power output,$\;{\;\dot{W}}_{\mathrm{net},\mathrm{btc}}$; (**c**) BCC thermal efficiency,$\;\eta_{\mathrm{bcc}}$.

The first optimization is done on the flow rates of BBC and BTC by conducting a two-dimensional parametric analysis with three-dimensional output, as presented in Figure 6. In this analysis, the mass flow rate of the BTC is varied from 20 to 50 kg s^−1^ in equal 5 kg s^−1^ increments, while BBC mass flow rate is varied from 6 to 18 kg s^−1^ in even 2 kg s^−1^ increments. The reason BTC flow rate is included in this parametric analysis: BTC is thermally coupled to the BBC. The input parameters of these two cycles are held constant and equal to the values in Table 2, except for part-flow ratio, ψ, and turbine expansion ratio, φ, of BTC. The base inputs for ψ and φ are taken as 2.51 and 0.68, respectively, as suggested by Utamura [21], and are optimized after the mass flow rates are determined.

There are two main outcomes of the conducted analysis in Figure 6. First, the thermal efficiency of BCC in Figure 6c shows a minima for the maximum flow rate of BTC at 50 kg s^−1^ and minimum flow rate of BBC at 6 kg s^−1^. The underlying reason for this result is the increase in the main compressor (MC) inlet temperature (State 1) of BTC. The increase in MC inlet temperature is directly proportional to BTC flow rate while being inversely proportional to BBC flow rate for a fixed effectiveness value of the BTC cooler, since the working fluid of BBC acts as a heat rejection medium for BTC. Note that the reason for utilizing CO_2_ as a working fluid in a closed-loop power cycle is to exploit the thermophysical properties of CO_2_ requiring minimum compression work in the vicinity of its critical temperature of 31.1 °C [48]. Therefore, the efficiency drops as the MC inlet temperature increases. This behavior is also prominent in Figure 6a, where topping cycle efficiency severely drops to 25%. Second, the BCC thermal efficiency exhibits another minima for the minimum flow rate of BTC at 20 kg s^−1^ and maximum flow rate of BBC at 18 kg s^−1^. Note that the BTC thermal efficiency reaches a maximum for this flow rate pair in Figure 6a, whereas BBC thermal efficiency is independent of the flow rates, owing to its fixed intensive thermodynamic properties defined by the KZD-1 steam turbine inlet temperature and constant ambient temperature. The underlying theory leading to this minima in BCC for this flow rate pair is the increase in the power generation share of BBC, which has a lower thermal efficiency compared to BTC.

The motivation for the flow rate selection procedure is to maximize BCC thermal efficiency while keeping the scale of BTC as small as possible to minimize the additional equipment cost. Therefore, the flow rate pair of 45 kg s^−1^ and 12 kg s^−1^ is selected for BTC and BBC, respectively. The mass flow rate of the total steam feeding the KZD-1 steam turbine increases from 19.45 kg s^−1^ to 31.45 kg s^−1^ with the addition of the biomass-derived dry steam and brings KZD-1 turbine close to its operation conditions in 2004 [34].

**Figure 7:**
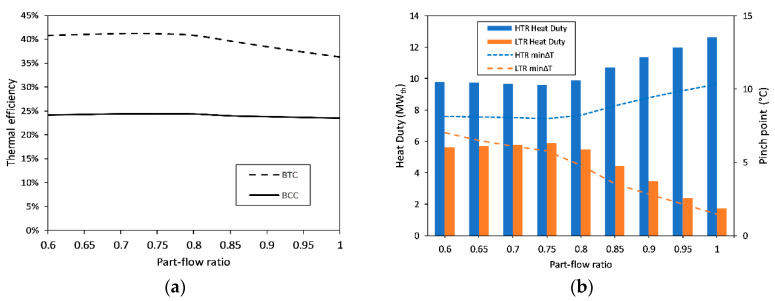
Optimization of part-flow ratio: (**a**) Variations in BTC and BCC thermal efficiencies with changing part-flow ratio; (**b**) Variations in the heat duties and pinch point of the recuperators with changing part-flow ratio.

**Figure 8:**
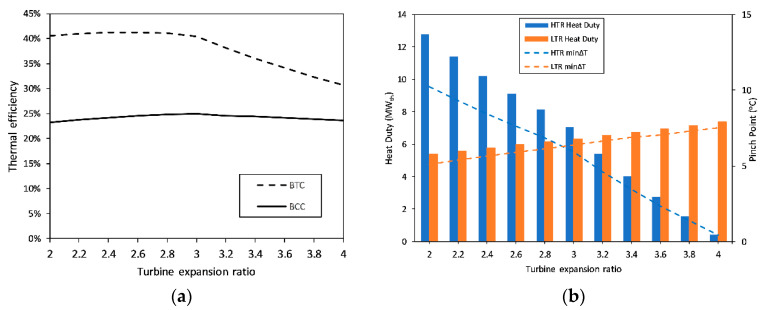
Optimization of turbine expansion ratio: (**a**) Variations in thermal efficiencies of BCC and BTC with changing turbine expansion ratio; (**b**) Variations in the heat duties and pinch point of the recuperators with changing turbine expansion ratio.

The two-characteristic parameters of BTC, namely, part-flow ratio, ψ, and turbine expansion ratio, φ, are optimized upon the decision of the flow rates. The part-flow ratio represents the ratio of the flow entering main compressor to the total flow; therefore, its unit value represents a simple-recuperated cycle. On the other hand, the turbine expansion ratio, φ, is the ratio of the turbine inlet pressure to turbine outlet pressure. These ratios not only affect the cycle-level performance parameters such as the thermal efficiency but also dictate the component-level indicators such as the heat duty and pinch point of the recuperators under the condition of constant effectiveness. Optimizations of these ratios are conducted, and the results are presented in Figure 7 and Figure 8. First, the part-flow ratio is varied from 0.6 to 1 in equal 0.05 increments, while all other inputs are kept constant and equal to the values in Table 2, except for the turbine expansion ratio, which is taken at its base value of 2.51 [21]. The part-flow ratio is selected as 0.75 to have the maximum BCC efficiency and minimum cumulative heat duty for the recuperators, while at least 5 °C pinch point is ensured. Upon the determination and fixing of ψ as 0.75, the turbine expansion ratio, φ, is varied from 2 to 4 in even 0.2 increments, while keeping all other inputs equal to their values in Table 2. Similarly, the optimum φ as chosen and finalized as 3 to have the maximum possible BCC thermal efficiency and minimum cumulative recuperator heat duty, where at least 5 °C pinch point is ensured for each recuperator.

**Figure 9:**
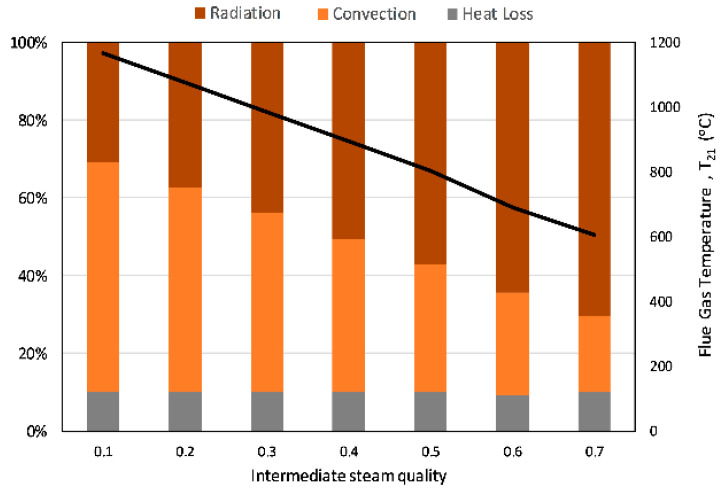
Heat transfer distribution and the flue gas temperature leaving the radiative section of the BHB (State 21) with varying intermediate steam quality.

The last optimization is done on the intermediate steam quality of the BBC (State 18). Since BHB design is discrete from the cycles, as discussed in Section 2.1.3., the intermediate steam quality does not affect the cycle parameters but is used as a boundary condition to determine the size of radiative and convective sections of the BHB. The preheated water at State 17 is heated by means of radiative combustion, to the intermediate steam quality in the radiative section of BHB. In addition to determining the size of the radiative and convective sections of BHB, this intermediate quality controls the temperature of the flue gas that is utilized in the convective section of the BHB. The results of the parametric study conducted on this intermediate steam quality imply, in Figure 9, that as more radiative heat is used to increase the intermediate steam quality, the temperature of the flue gas decreases. In order to keep the flue gas temperature below 1000 °C, as suggested by Strzalka et al. [49], and have a typical heat distribution in the range of radiation chamber (45–55%; convective Section 45–25%; stack and other heat losses: l0–20%, as suggested by Stehlík et al. [50]), the intermediate steam quality is chosen to be 0.4. Note that the retention of a flue gas temperature below 1000 °C is achieved either by increasing the excess air ratio [32] or recirculating the flue gas inside the furnace [49] in the literature. In this article, temperature control of the flue gas is controlled through the intermediate steam quality at State 18. However, a novel biomass heater-boiler design, to supply heat to two power cycles with different working fluids, is beyond the scope of this article and may require further investigation.

## 3. Results and Discussion

**Figure 10:**
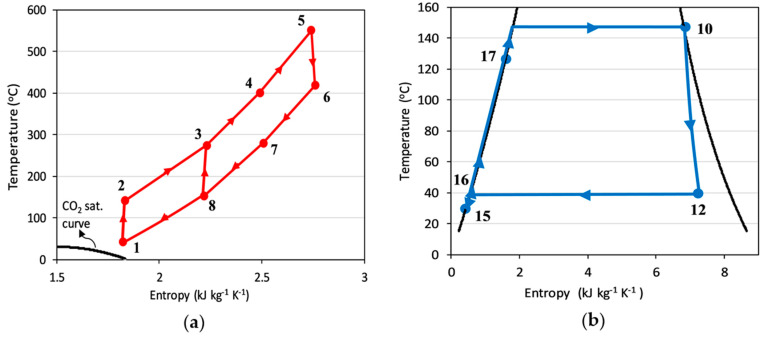
T-s diagrams of the thermodynamic cycles used in hybridization of KZD-1 GEPP: (**a**) BTC; (**b**) BBC.

The energetic results of the cycles used in the hypothetical hybridization scenario of KZD-1 GEPP are presented in Table 4. The design parameters are provided in Table 2 for reproducibility of the results. T-s diagrams of BTC and BBC are shown in Figure 10.

**Figure 11:**
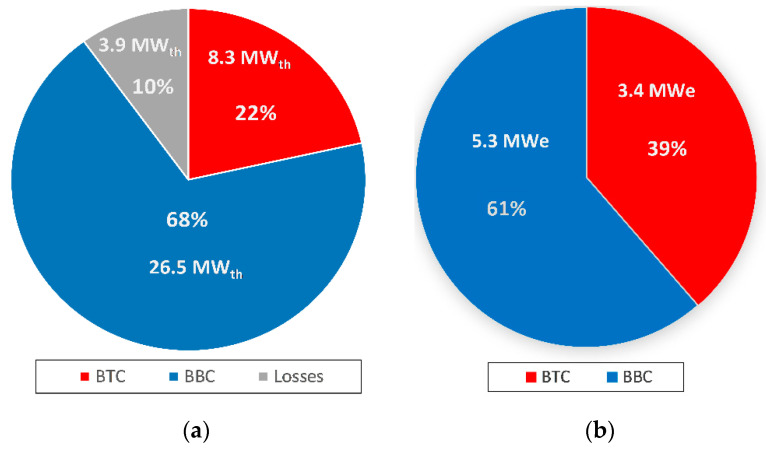
Heat and power distribution between the cycles: (**a**) Allocation of biomass energy; (**b**) Net power distribution.

The thermal efficiency of the standalone part-flow sCO_2_ cycle with the same turbine inlet temperature (TIT) of 550 °C, as in this article, is reported to be around 46.5% in the literature [22]. In this work, it is expected that the efficiency of topping the sCO_2_ cycles will be penalized slightly to maximize the performance of the combined cycle. However, the efficiency penalty of more than 5% exceeds this expectation. For example, the part-flow sCO_2_ topping cycle in the study of Manente and Lazzaretto [32] has 44.2% thermal efficiency for the same maximum turbine inlet conditions as given in this article. The underlying theory leading to an overly penalized BTC efficiency in this article is the deviation from optimum compressor inlet conditions at State 1. The sCO_2_ cycles take advantage of the minimal compression work of the working fluid CO_2_ in the vicinity of its critical temperature of 31.8 °C. Note that the temperature of State 1 in this paper is 42.9 °C, which causes the T-s diagram of the topping cycle to shift slightly right of the saturation curve of CO_2_ and ultimately decreases the cycle efficiency by about 5% compared to results in the literature. The reason for the deviation from the optimum CO_2_ compression inlet temperature is that the rejected heat of BTC is recovered in BCC using the coupling heat exchanger, cooler. As the flowrate of the BBC increases, the temperature of the CO_2_ at the hot side outlet of cooler (State 1) increases under a fixed effectiveness value of the cooler. Although this process penalizes the efficiency of BTC by around 5%, it allows for the utilization of 5 MW thermal heat in the bottoming cycle. In fact, the scale of the topping cycle should be kept as small as possible to use the existing steam turbine of KZD-1 by BBC to the fullest extent. Therefore, the efficiency drop in BTC is diluted in the combined cycle due to the utilization of a significant portion of the biomass-derived heat in BCC, as shown in Figure 11. As a comparison, the net power distribution in the work of Manente and Lazzaretto [32] favors the topping cycle by 90% (topping) to 10% (bottoming), while the bottoming cycle is favored in this article by 61% (bottoming) to 39% (topping).

The thermal efficiency of the single-flash GEPPs with a TIT of 150 °C lies in the range of 15–18% in the literature [51]. Although the 16.9% thermal efficiency of BBC falls in this range, 24.9% thermal efficiency of BCC can be considered to be penalized itself, due to its favoring BBC in biomass energy utilization, as seen in Figure 11a. There is no similar combined cycle configuration in the literature where sCO_2_ and steam Rankine cycles are utilized in a cascaded manner, as in this article, to allow for a comparison of BCC thermal efficiency. However, Jiang et al. reported a hybrid solar thermal–EGS power plant using CO_2_ as a working fluid as reaching 21.93% and 22.44%, respectively, for TITs of 500 °C and 600 °C, which is in relatively good agreement with the found BCC efficiency [15]. On the other hand, Manente and Lazzaretto reported their biomass-to-electricity conversion efficiency as 34.3% for their combined cycle [32]. Despite their topping cycle being a part-flow sCO_2_ cycle with the same turbine inlet conditions as in this article, it should be noted that their bottoming cycle is a simple recuperated sCO_2_ cycle with a TIT of 313.9 °C, while the TIT of the bottoming steam Rankine cycle in this article is restricted by the operational TIT of KZD-1 GEPP at 146.9 °C. Therefore, a lower biomass-to-electricity conversion efficiency is expected in this article, compared to their 34.3% conversion efficiency.

Within the scope of this article, the mass flow rate of the biomass fuel in BHB is adjusted so that the temperature of the exhaust flue gas at air preheater outlet (State 23) is limited to the minimum allowable temperature of 110 °C to prevent dew point condensation after the demanded heat is supplied to the combined cycle [31]. The results of the BHB parameters, including flue gas mass flow rate, flue gas temperature leaving the radiative section of BHB at State 21, flue gas composition, calculated adiabatic flame temperature, and the BHB efficiency, are presented in Table 5. Based on the mass flow rate of the biomass fuel, the biomass-to-electricity conversion efficiency is calculated as 22.4% using Equation (12).

**Figure 12:**
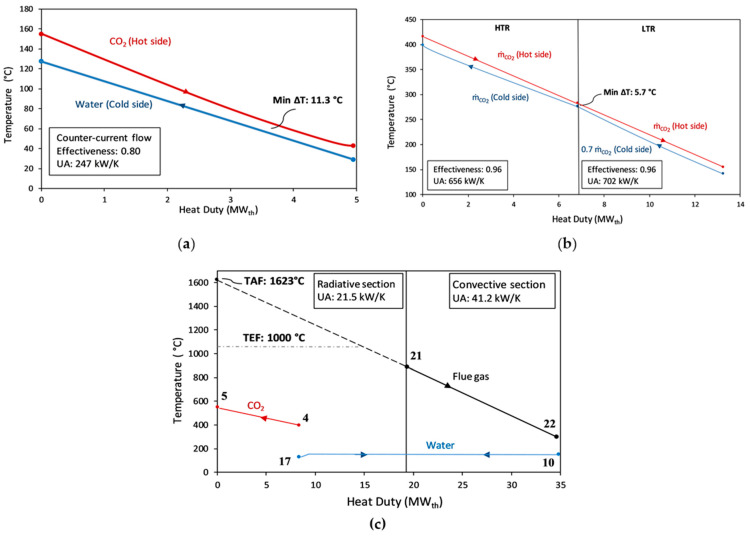
Q-T diagram of the heat exchangers. (**a**) Cooler; (**b**) Recuperators; (**c**) Radiative-convective counter-current heater-boiler (BHB) with assumed TEF = 1000 °C.

Even though this paper lacks economic and exergy analysis, preliminary remarks can be made on the Q-T diagrams of the heat exchangers presented in Figure 12. For the recuperators, it is shown that no pinch problem exists, and the minimum temperature difference between two streams is kept larger than 5 °C, with an effectiveness of 96%. Although it is expected that the recuperators will have a good temperature match between the cold and hot flows, as they have the same working fluid on both sides, the good temperature match for the cooler is promising in terms of its low potential for exergy destruction, and stems from the fact that water is sensibly heated through the cooler. Since the cooler acts as a thermal coupling mechanism between BTC and BBC, it can be suggested that a good synergy between the two intrinsically different cycles is achievable.

The heat addition to BTC in this article is only possible between state points 4 and 5 in Figure 11a owing to the highly recuperative characteristics of the sCO_2_ cycles. Thus, heating below a certain temperature, i.e., 400 °C in this article, cannot be utilized in the topping sCO_2_ cycle without changing its layout or adding additional heat exchangers. In order to overcome this problem, sCO_2_ cycles are generally utilized in the literature in a cascaded manner, as mentioned in Section 1 and Section 2. Since a flue gas heat below 400 °C is utilized in BBC through the convective section of BHB, the problem of having a complex sCO_2_ cycle layout or adding another sCO_2_ cycle as a bottoming cycle is resolved. Finally, UA values (commonly known as conductance and expressed in units of kW K^−1^) for each heat exchanger are supplied as a preliminary economic indicator. It is assumed that the equipment cost scale is within the UA value; recuperators appear to be the heat exchanger units requiring most of the investment costs [44,47]. 

## 4. Conclusions

In this article, the underperforming KZD-1 GEPP is theoretically hybridized using a biomass-driven sCO_2_ topping and steam Rankine bottoming cycles where locally sourced olive residue is used as a biomass fuel source. While a topping sCO_2_ cycle is specifically chosen due to its potential for flexible electricity generation, as a first step to develop this novel hybridization scheme, only the steady-state design conditions of hybridization are modeled in this work. The proposed working fluid mass flow rates for topping and bottoming cycles, i.e., 45 kg s^−1^ and 12 kg s^−1^, respectively, can represent the nominal case. For off-design scenarios, these flow rates can be downscaled proportionally by controlling the combusted biomass fuel flow, such that the intensive thermodynamic properties remain close to their design values. Although a decrease in both thermal efficiency and power generation can occur for off-design calculations, the scenarios for hourly fluctuations or seasonal variations can be investigated in future work.

The hybridization increases the nominal flow rate of the steam feeding the KZD-1 turbine from 19.45 s^−1^ to 31.45 s^−1^ and brings the steam turbine closer to its operating conditions reported in 2004 [34]. Then, 3.4 MWe and 5.3 MWe additional net powers are generated through the topping and bottoming cycles, with 40.1% and 16.9% thermal efficiencies, respectively. The combined cycle composed of the combination of topping and bottoming cycle has a thermal efficiency of 24.9% and net power generation of 8.7 MWe. Biomass to electricity conversion efficiency is calculated as 22.4% for a fuel consumption rate of 2.2 kg s^−1^. Despite the penalties in terms of topping cycle thermal efficiency and biomass-to-electricity conversion efficiency compared to the literature, the motivation in the hybridization scenario in this article is using the existing infrastructure of KZD-1 GEPP to the fullest extent by keeping the topping cycle and additional investments costs as small as possible while retaining the maximum possible efficiency. In this context, the goal of sharing existing components in hybrid power plant layouts is arguably achieved [47]. Moreover, the high-temperature heat addition problem of sCO_2_ cycles is resolved by utilizing a flue gas heat under 400 °C in the bottoming cycle. Consequently, the need to add a bottoming sCO_2_ cycle or have a complex sCO_2_ cycle layout is avoided.

The hybridization of an existing GEPP in this article is achieved by the addition of a sCO_2_ cycle, a biomass heater boiler, and a dry cooling system. Although reliable cost correlation parameters of conventional systems, such as boilers or dry cooling systems, are present in the literature for economic analysis, the equipment costs of next-generation sCO_2_ power cycles that have not been commercialized yet remain unknown. As a rough estimation, Wright et al. consider the cost of all heat exchangers in the sCO_2_ cycle (recuperators, primary heat exchangers, preheaters, etc.) as about 50% of the total system costs [52]. Even if a complete economic analysis is not incorporated in this article, UA values of the heat exchangers are supplied in Figure 12 as an economic indicator, and sCO_2_ recuperators seem to be the components that would account for most of the additional investment costs of such a hybridization.

Although this paper considers a KZD-1 GEPP hybridization scenario as a case study, the results can be adapted to different locations, e.g., to a single-flash GEPP in Philippines using a rice husk as the fuel source. In fact, there would be no need to create a hypothetical bottoming steam Rankine cycle, as in this article, if such a single-flash GEPP works in the cyclic mode contrary to the open-cycle case of KZD-1 GEPP. In that case, the existing closed-loop steam Rankine cycle can be used as the bottoming cycle and offer even better thermal and cost efficiency, since the existing unused cooling system can be utilized and the need for an additional dry cooling system is eliminated.

## Figures and Tables

**Table 1:** Analysis of the biomass fuel.

Parameter	
Proximate analysis (wt.%, dry basis)	
Volatile matter	83.9
Fixed Carbon ^a^	14.2
Ash	1.9
Moisture content (wt.%, as received)	
Moisture	7.5
Ultimate analysis (wt.%, dry ash free)	
C	51.5
H	6.2
N	0.7
S	-
O ^a^	41.6
Calorific value	
Higher heating value (dry basis) ^b^ (MJ kg^−1^)	20.5
Lower heating value (wet basis) ^c^ (MJ kg^−1^)	17.5

^a^ Calculated by difference ^b^ Calculated by empirical correlation [37] ^c^. Calculated by empirical correlation [32].

**Table 2:** Constants and inputs for the proposed hybrid plant.

Input	Value	Unit	Description	Source/Comment
BTC				
η_comp_	90	%	Isentropic efficiency of the compressors	[21,32]
η_turb_	93	%	Isentropic efficiency of the turbine	[21,31,44]
T_turb_	550	°C	Turbine inlet temperature	[22,32,42,45]
P_turb_	20	MPa	Turbine inlet pressure	[21]
φ	3	-	Expansion ratio of the CO_2_ turbine, P_5_/P_6_	Optimized parameter [21].
ψ	0.75	-	Part-flow ratio, ṁ_1_/ṁ_8_	Optimized parameter [21].
ε_recup_	96	%	Effectiveness of the recuperators	[21,39,46]
ε_cooler_	80	%	Effectiveness of the cooler	[21,46]
ΔP_hot,recup_	0.03	MPa	Pressure drop at hot side of recuperators	[21,47]
ΔP_cold,recup_	0.22	MPa	Pressure drop at cold side of recuperators	[21,47]
ΔP_hot,cooler_	0.6	MPa	Pressure drop at hot side of cooler	[21,47]
ΔP_cold,cooler_	0.1	MPa	Pressure drop at cold side of cooler	[21,47]
ΔP_bhb,co2_	0.24	MPa	Pressure drop for CO_2_ inside BHB	[21,47]
BBC				
η_turb,bot_	80	%	Isentropic efficiency of the steam turbine	Assumption. See Section 2.1.1
η_pump_	80	%	Isentropic efficiency of the pumps	Generic value.
η_air comp_	90	%	Isentropic efficiency of the air compressor	Generic value.
T_turb,bot_	146.9	°C	Turbine inlet temperature	Operational KZD-1 data.
P_turb_	0.438	MPa	Turbine inlet pressure	Operational KZD-1 data.
ΔP_bhb,water_	0.01	MPa	Pressure drop for water inside BHB	Generic value.
BHB				
λ	1.5	-	Excess air ratio	[32]
TEF	1000	°C	Effective temperature of radiation	[32]
Rad. loss	5	%	Heat loss in the radiative section of BHB	[32]
ε_air preheater_	80	%	Effectiveness of the air preheater	[46]
T_air,in_	20	°C	Air inlet temperature to air preheater	[32]

**Table 3:** BHB verification results based on the model of Manente and Lazzaretto [32].

Inputs		Outputs					
λ	T_air_ (°C)	T_flue gas_ (°C)	m_flue gas_ (kg s^−1^)	X_CO2_	X_H2O_	X_N2_	X_O2_
Manente and Lazzaretto [32]							
1.5	20	1405	8.225	0.1826	0.0754	0.6738	0.0682
	100	1457					
2.37	20	1000	12.41	0.1211	0.0499	0.7053	0.1237
2.56	100	1000	13.33	0.1127	0.0465	0.7095	0.1313
Verification model							
1.5	20	1407	8.273	0.1820	0.0749	0.6752	0.0679
	100	1458.6					
2.37	20	999.9	12.49	0.1230	0.0496	0.7065	0.1209
2.56	100	1000.7	13.41	0.1125	0.0462	0.7107	0.1306
Error: (Verif. Model—Ref.)/Ref.							
		0.1%	0.6%	–0.3%	–0.7%	0.2%	–0.4%
		0.1%					
		0.0%	0.6%	1.6%	–0.6%	0.2%	–2.3%
		0.1%	0.6%	–0.2%	–0.6%	0.2%	–0.5%

**Table 4:** Energetic results of the cycles used in hybridization of KZD-1 GEPP.

Cycle	ṁ kg s^−1^	η_th_ %	Ẇ_net_ MWe
Topping part-flow sCO_2_ (BTC) Bottoming steam Rankine (BBC)	45	40.1	3.4
	12	16.9	5.3
Combined (BCC)	-	24.9	8.7

**Table 5:** Radiative-convective counter-current heater-boiler (BHB) results.

η_bhb_	ṁ_fuel_ (kg s^−1^)	ṁ_flue gas_ (kg s^−1^)	TAF (°C)	T_21_ (°C)	X_CO2_	X_H2O_	X_N2_	X_O2_
0.90	2.2	21.5	1623	890.6	0.1775	0.0623	0.6909	0.0693

## Data Availability

The data presented in this study are available on request from the corresponding author.

## Abbreviations/Nomenclature

b2e	biomass to electricity
BBC	biomass bottoming cycle
BCC	biomass combined cycle
BHB	biomass heater boiler
BTC	biomass topping cycle
CSP	concentrated solar power
DC	direct contact
DSG	direct steam generation
EGS	enhanced geothermal system
GEPP	geothermal electric power plant
HHV	higher heating value
HP	lower heating value
HTR	High-temperature recuperator
KZD-1	Kızıldere-1
KZD-2	Kızıldere-2
LCOE	levelized cost of electricity
LHV	lower heating value
LP	low pressure
LTR	Low-temperature recuperator
MC	main compressor
NGC	non-condensable gas
OR	olive residue
ORC	organic Rankine cycle
PTC	parabolic trough collector
PV	photovoltaic
RC	recompressor
sCO_2_	supercritical CO_2_
TAF	adiabatic flame temperature
tCO_2_	transcritical CO_2_
TEF	effective temperature of radiation
TIT	turbine inlet temperature
WCT	wet cooling tower
WHR	waste heat recovery
λ	excess air ratio
